# Toll-Like Receptor 4 Signaling Pathway Mediates Inhalant Organic Dust-Induced Bone Loss

**DOI:** 10.1371/journal.pone.0158735

**Published:** 2016-08-01

**Authors:** Elizabeth Staab, Geoffrey M. Thiele, Dillon Clarey, Todd A. Wyatt, Debra J. Romberger, Adam D. Wells, Anand Dusad, Dong Wang, Lynell W. Klassen, Ted R. Mikuls, Michael J. Duryee, Jill A. Poole

**Affiliations:** 1 Pulmonary, Critical Care, Sleep & Allergy Division, Department of Internal Medicine, University of Nebraska Medical Center, Omaha, NE, United States of America; 2 Veterans Affairs Nebraska-Western Iowa Health Care System, Omaha, NE, United States of America; 3 Rheumatology Division, Department of Internal Medicine, University of Nebraska Medical Center, Omaha, NE, United States of America; 4 Department of Environmental, Agricultural, and Occupational Health, University of Nebraska Medical Center, Omaha, NE, United States of America; Shool of Pharmaceutical Sciences, Sun Yet-Sen University, 132 Easy Cycle at University City, CHINA

## Abstract

Agriculture workers have increased rates of airway and skeletal disease. Inhalant exposure to agricultural organic dust extract (ODE) induces bone deterioration in mice; yet, mechanisms underlying lung-bone crosstalk remain unclear. Because Toll-like receptor 2 (TLR2) and TLR4 are important in mediating the airway consequences of ODE, this study investigated their role in regulating bone responses. First, swine facility ODE stimulated wild-type (WT) bone marrow macrophages to form osteoclasts, and this finding was inhibited in TLR4 knock-out (KO), but not TLR2 KO cells. Next, using an established intranasal inhalation exposure model, WT, TLR2 KO and TLR4 KO mice were treated daily with ODE or saline for 3 weeks. ODE-induced airway neutrophil influx and cytokine/chemokine release were similarly reduced in TLR2 and TLR4 KO animals as compared to WT mice. Utilizing micro-computed tomography (CT), analysis of tibia showed loss of bone mineral density, volume and deterioration of bone micro-architecture and mechanical strength induced by ODE in WT mice were significantly reduced in TLR4 but not TLR2 KO animals. Bone marrow osteoclast precursor cell populations were analyzed by flow cytometry from exposed animals. In WT animals, exposure to inhalant ODE increased osteoclast precursor cell populations as compared to saline, an effect that was reduced in TLR4 but not TLR2 KO mice. These results show that TLR2 and TLR4 pathways mediate ODE-induced airway inflammation, but bone deterioration consequences following inhalant ODE treatment is strongly dependent upon TLR4. Thus, the TLR4 signaling pathway appears critical in regulating the lung-bone inflammatory axis to microbial component-enriched organic dust exposures.

## Introduction

Bone diseases, particularly osteoporosis and fracture, are recognized as debilitating systemic features of several types of chronic inflammatory lung diseases including chronic obstructive pulmonary disease (COPD), asthma, cystic fibrosis, and post-lung transplant [1–5]. In COPD and asthma, studies demonstrate that low bone mineral density and osteoporosis can occur independently of established osteoporosis risk factors including low body mass index, gender, age, sedentary life-style, cigarette smoking, nutritional status, and medications [2,3]. Approximately two-thirds of agriculture workers develop respiratory diseases (e.g. rhinosinusitis, asthma, chronic bronchitis, COPD) [6–9], and nearly 90% suffer from musculoskeletal disease, including high fracture rates [10–12]. Recently, we established an animal model demonstrating that intranasal inhalation of organic dust extract (ODE) collected from large animal (swine) confinement facilities and its microbial components induce systemic bone loss [13]. Whereas vitamin D supplementation was demonstrated to protect against the adverse bone health consequences induced by inhalant exposures [14], the underlying mechanisms that regulate the lung-bone inflammatory axis are not known.

Agriculture-related organic dust is a complex mixture enriched with an abundance of gram positive and gram negative microbial cell wall components [9]. Although endotoxin is an important inflammatory component present in organic dust [15], the association with endotoxin exposure and human airway disease manifestations have been inconsistent [16,17]. Recent studies have described a predominance of gram positive bacteria in agriculture dust samples [18–20], and genetic polymorphisms in Toll-like receptor 2 (TLR2) gene have been associated with airway disease in swine confinement workers [21]. In animal studies, TLR2, which recognizes peptidoglycans, lipoteichoic acid and lipopoproteins associated with gram positive bacteria, and TLR4, which recognizes gram negative endotoxin, have been both implicated in regulating organic dust-induced lung inflammation [22–24]. Specifically, in mice deficient in TLR2 or TLR4 and challenged with organic dust environments or extracts, it has been demonstrated there is up to a 50% reduction in various airway inflammatory consequences [22,24]. However, their respective role in mediating systemic bone consequences has not been previously described. This is an important knowledge gap recognizing in as much as TLR ligands have been shown by others to increase osteoclastogenesis in a dose-dependent manner [25–28]. Although it is well recognized that osteoclasts express TLRs, the potential impact of inhalant ODE exposures on potentially regulating osteoclast progenitor cell populations is not known.

Therefore, this study is based on the hypothesis that TLR2 and TLR4 signaling pathways are equally important in regulating the susceptibility to systemic bone consequences induced by inhaled swine confinement ODE exposures. To test this hypothesis, an in vitro osteoclastogenesis assay was first utilized in proof-of-concept studies to delineate the role of TLR2 and TLR4 signaling pathways in promoting ODE-mediated osteoclast development. Next, TLR2 and TLR4 knock out (KO) animals were subjected to intranasal inhalant ODE exposures and airway inflammatory, systemic bone disease, and bone marrow osteoclast precursor population consequences were investigated and compared to wild type (WT) control mice.

## Methods

### Organic dust extract

Aqueous ODE was prepared as previously described [29]. Briefly, settled dust was collected from horizontal surfaces (~3 feet above floor level) of swine confinement feeding operations, with knowledge and permission granted by the swine confinement owners. These operations are located in Colfax County, Nebraska (population density approximates 25 people per square mile). Dust (1 gm) was incubated in sterile Hank’s Balanced Salt Solution (10 ml; Sigma, St. Louis, MO) at room temperature for 1 hour, centrifuged for 60 min at 2000 x *g*, and the final supernatant was filter sterilized (0.22 μm), a process that also removes coarse particles. Endotoxin concentrations in 100% ODE ranged from 1240–1400 EU/ml as determined using the limulus amebocyte lysate assay (Sigma). Muramic acid levels were previously determined by mass spectrometry to be approximately 70 ng/mg [14]; muramic acid is a molecular component of bacterial cell wall peptidoglycans. Stock ODE was diluted to a final concentration (vol/vol) of 0.5% for cell culture studies and 12.5% for animal studies in sterile phosphate buffered saline (PBS; pH: 7.4; diluent). ODE 12.5% has been previously shown to elicit optimal experimental outcomes in mice and is well tolerated [30].

### Animals

This study was carried out in strict accordance with the recommendations in the Guide for the Care and Use of Laboratory Animals of the National Institutes of Health. All the animal procedures were approved by the Institutional Animal Care and Use Committee at the University of Nebraska Medical Center (UNMC) and were in accordance with the NIH guidelines for the use of rodents (UNMC protocol number 10-054-07). Mice were housed in our Durham Research Center Building II animal facility. The UNMC Staff Veterinarians and technicians supervise the facility. Mice were housed in group cages (up to 5/cage) and fed commercial rodent chow and water ad libitum. The facility operates on a 12 hour light/12 hour dark cycle. TLR2 gene knockout (KO) and TLR4 gene KO mice on C57BL/6 background were provided by Dr. S. Akira (Osaka, Japan). C57BL/6 mice purchased from The Jackson Laboratory (Bar Harbor, ME) were used as wild-type (WT) controls. Male mice, between 7–16 weeks, were used for all studies.

### In vitro murine osteoclastogenesis assay

An *in vitro* murine cell osteoclastogenesis assay using receptor activator of NF-κB ligand (RANKL)-pretreated bone marrow macrophages, developed by others to investigate the role of endotoxin in osteoclast biology [26] was employed to study the role of TLR2- and TLR4- dependent pathways in promoting ODE-induced osteoclastogenesis. Briefly, bone marrow cells were isolated from long bones of WT, TLR2 KO, and TLR4 KO mice, cell solution passed through nylon mesh (70 μM; Thermo Fisher Scientific, Waltham, MA), and cell pellets were briefly resuspended in sterile water to lyse red blood cells before resuspending in PBS. After centrifugation, bone marrow cells were cultured in Minimal Essential Medium α (Thermo Fisher Scientific) containing 10% heat-inactivated fetal bovine serum, 2 mM L-glutamine, and penicillin/streptomycin in the presence of M-CSF (44 ng/ml; eBioscience, San Diego, CA) and RANKL (100 ng/ml; PeproTech, Rocky Hill, NJ) for 3 days. On day 3, the bone marrow-derived macrophages were washed, re-cultured in M-CSF and RANKL, and stimulated with ODE (0.5%), lipopolysaccharide from *Escherichia coli* (O55:B5) (LPS/TLR4 agonist, 10 ng/ml; Sigma), or *Staphylococcus aureus* peptidoglycan (PGN/TLR2 agonist, 1 μg/ml; Sigma) for an additional 3 days. Osteoclast formation was assessed by mRANKL expression by flow cytometry analysis [31–33]. Briefly, cells were stained with monoclonal antibody directed against mRANKL and the appropriate isotype control (Thermo Fisher Scientific). Osteoclast development was determined by percentage of mRANKL expression as determined by rightward shift in fluorescence as compared to control.

### Exposure animal model

The established intranasal (i.n.) inhalation exposure animal model was utilized whereby mice were lightly sedated under isoflurane and received treatment with 50 μl of sterile saline (PBS) or 12.5% ODE (50 μl volume) daily for 3 weeks [22,30,34]. Animals were euthanized and measurements taken 5 hours following the final inhalation treatment. WT saline- and ODE- treated mice were compared in side-by-side experimental runs with TLR2 KO and TLR4 KO saline- and ODE- treated animals. No animals displayed suffering or distress, and moreover, no animals died prior to the experimental endpoints.

#### Bronchoalveolar lavage fluid neutrophil and cytokine/chemokine analysis

Bronchoalveolar lavage fluid was collected using 3 x 1 ml PBS. Total cell numbers from pooled lavages were enumerated and neutrophils determined from cytospin-prepared slides (cytopro cytocentrifuge, ELITechGroup, Logan, UT) stained with DiffQuick (Siemens, Newark, DE). Interleukin (IL)-6, tumor necrosis factor-alpha (TNF-α), IL-1β, keratinocyte chemoattractant (CXCL1; a murine neutrophil chemoattractant), and macrophage inflammatory protein-2 (CXCL2; a murine neutrophil chemoattractant) were quantitated in the cell-free supernatant of the first lavage by ELISA kits (R&D systems) with sensitivities of 1.8, 7.2, 4.8, 2.0, and 1.5 pg/ml, respectively.

#### Serum

At time of euthanization, whole blood was collected from mice from the axillary artery, placed in BD Microtainer Tubes (Becton, Dickinson and Company, Franklin Lakes, NJ), centrifuged, and cell-free serum collected. Serum TNF-α, IL-6, IL-1β, and IL-17 were quantified according to the manufacturer’s instruction using a Quantikine enzyme-linked assay kit (R&D Systems). Serum levels of tartrate-resistant acid phosphatase 5b (TRACP 5b) were also quantified according the manufacturer’s instruction using the MouseTRAP assay from Immunodiagnostic Systems Inc (Gaithersburg, MD) with sensitivity of 0.1 U/L.

### Micro-CT analysis of tibias

The right hind limbs from exposed mice were excised, processed, and prepared for micro-computed tomography (CT) scanning and analysis as previously described [13,14,35]. Briefly, the proximal tibia was scanned using high-resolution micro-CT (Skyscan 1172; Skyscan, Aartselaar, Belgium) with images acquired at a resolution of 6.07 μm, source set at 48 kV and 187 μ with 0.5-mm-thick aluminum filter with an exposure time of 620 ms. Scanning performed at 0.4° intervals, and 6 average frames were obtained for each rotation. NRecon (Skyscan) software was used to reconstruct scanned images, and analysis was conducted on stacked reconstructed images using CTAn (Skyscan) software as previously described [35]. To ensure proper orientation along the longitudinal axis, growth plates were identified as the reference point and tibial position was corrected using Dataviewer (Skyscan). Analysis started at 75 slices distal to the reference point, and mineralized cartilage was excluded from analysis. Final analysis was conducted on an interpolated region of interest (manually drawn to exclude the cortical shell) from a volume of interest of 1.82 mm distance (300 × 6.07 μm; 300 slides). CT-Vox and CT-Vol software (Skyscan) were used to construct three-dimensional (3D) images.

Standard 3D parameters were measured for the trabecular bone in the proximal tibial metaphysis: bone mineral density, specific bone surface (bone surface to bone volume ratio), trabecular thickness, trabecular separation, trabecular pattern factor (measurement of bone structural connectivity), and polar moment of inertia. Methods to calculate these bone parameters are described on the Skyscan website (www.skyscan.be). Bone mineral density and trabecular thickness decrease with quantitative bone loss, whereas trabecular separation, specific bone surface area, and trabecular pattern factor increase with bone deterioration. Polar moment of inertia represents the geometric index of bone strength to resist torsion, with lower values suggestive of lower bone quality [36]. To compare bone parameter findings across animal strains (i.e. WT, TLR2 KO, and TLR4 KO), the percent change induced by ODE treatments (difference between ODE and saline treatment groups divided by saline control multiplied by 100) were compiled from three independent studies of 2–3 mice per study (N = 6–9).

#### Phenotyping bone marrow for osteoclast precursor populations

Bone marrow cells from the long bone of hind limbs (i.e. femur, tibia) were flushed with 10 mL of sterile PBS and passed through a nylon mesh (70 μM; Thermo Fisher Scientific Waltham, MA) to remove any large fragments. Red blood cells were lysed briefly suspending in cold sterile water and subsequently resuspended in PBS. Following centrifugation, the remaining cells were re-suspended in 0.1% BSA in PBS for staining with a LIVE/DEAD Fixable Violet Dead Cell Stain kit (Life Technologies, Carlsbad, CA) that was used to assess cell viabilities. There were no differences in cell viability between saline and ODE-treated groups or between WT and KO mice (data not shown). After washing, bone marrow cells from each animal were stained with monoclonal antibodies (mAb) directed against T-cell lineage: CD3, B-cell lineage: B220/CD45R, monocyte/macrophage lineage: Mac-1/CD11b, and against markers of progenitor cells: c-fms/CD115, c-kit/CD117, and CD27 (BD Biosciences, San Jose, CA). Parallel cell preparations were treated with appropriate isotype control mAb. Compensation was performed with antibody capture beads (eBiosciences, San Diego, CA) stained separately with each individual mAb used in test samples.

The gating strategy for osteoclast precursor (OCP) populations utilizes several published, step-wise approaches to define bone marrow OCPs [37–40]. After exclusion of debris and dead cells, initial gating was on triple negative (TN: CD45R^-^, CD3^-^, CD11b^lo^) cell population because most of the osteoclastogenic activity of total bone marrow resides in the TN fraction [37–40]. Next, CD115 and CD117 were utilized to further dissect the TN population. CD115 is the receptor for M-CSF and CD117 (c-kit) is the receptor for stem cell factor. Both are markers of early hematopoietic lineage as well as OCPs. Jacquin and colleagues [37] demonstrated that most of the early osteoclastogenic activity of the TN bone marrow fraction is contained within the TN CD115^+^CD117^+^ population. Xiao and colleagues [38] subsequently demonstrated that CD27 expression on TN CD115^+^CD117^+^ populations further discriminates cells that are highly enriched for osteoclastogenic potential.

### Statistical methods

Data are presented as the mean ± standard error of mean (SEM). To detect significant changes between groups, a one-way analysis of variance (ANOVA) was utilized and a post hoc test (Tukey/LSD) was performed to account for multiple comparisons if the *p* value was < 0.05. All statistical analysis was performed using SPSS software (SPSS, Chicago, IL, USA) and statistical significance accepted at *p* < 0.05.

## Results

### ODE-induced osteoclastogenesis is dependent on the TLR4, but not the TLR2, signaling pathway *in vitro*

**Fig 1:**
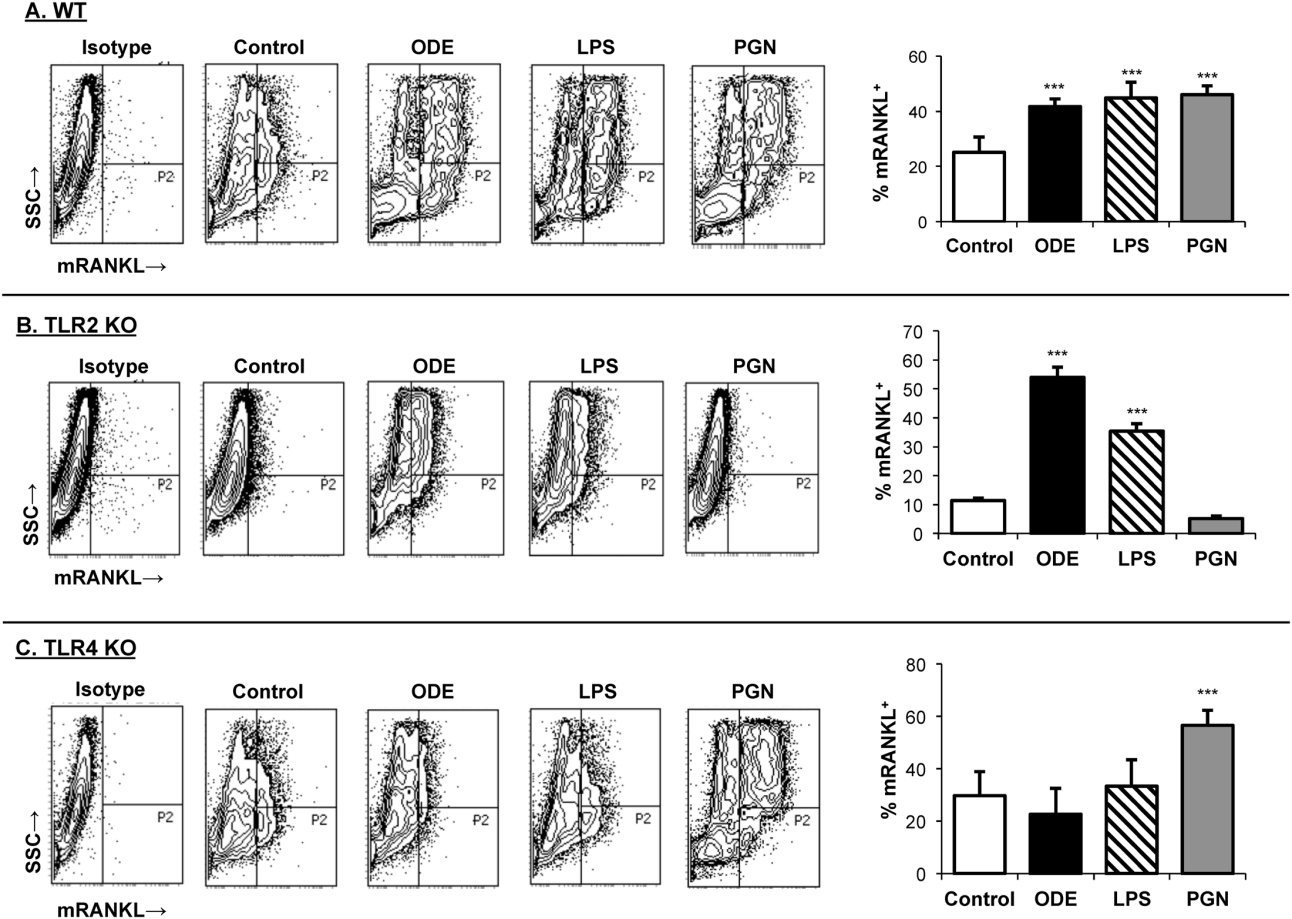
Organic dust extract (ODE) promotes osteoclastogenesis *in vitro* with WT and TLR2 KO, but not TLR4 KO, bone marrow macrophages. Bone marrow derived cells from C57BL/6 WT mice (**A, top panel**), TLR2 KO mice (**B, middle panel**), and TLR4 KO (**C, bottom panel**) were cultured with M-CSF and RANKL and ± ODE (0.5%). In side-by-side experiments, cells were also cultured with a TLR4 agonist (lipopolysaccharide; LPS; 10 ng) or TLR2 agonist (peptidoglycan; PGN; 1 μg) as additional controls. Membrane receptor activator of NF-κB ligand (mRANKL) expression was determined by flow cytometry analysis. A representative contour plot from each experimental group is shown with a rightward shift demonstrating gating of positive mRANKL expression after exclusion of debris. Bar graphs show the mean with standard error bars of percentage of mRANKL expression (P2 gate). N = 6/group from three separate studies ran in duplicate. Statistical significance denoted by asterisks (***p<0.001) vs. unstimulated control.

In these studies, osteoclastogenesis was determined by mRANKL expression from murine bone marrow cells pre-treated with M-CSF and RANKL for 3 days and then subsequently stimulated with ODE, LPS (TLR4 agonist), or PGN (TLR2 agonist) for an additional 3 days. Referent to saline, ODE, LPS, and PGN treatments significantly increased mRANKL expression as compared to unstimulated control (p<0.001; Fig 1A) from WT derived bone marrow cells. ODE treatment similarly promoted osteoclastogenesis from TLR2 KO bone marrow cells (Fig 1B). In contrast, osteoclast development was inhibited in TLR4 KO bone marrow cells treated with ODE (Fig 1C). Note that PGN promoted osteoclastogenesis with TLR4 KO cells, indicating that the inhibitory effect demonstrated for ODE was not explained by an inherent defect in the osteoclastogenic ability of TLR4 KO cells. These studies demonstrate that TLR4, but not TLR2, is sufficient to promote ODE-induced osteoclastogenesis from M-CSF and RANKL-pretreated bone marrow macrophages *in vitro*.

### Repetitive inhalant ODE-induced airway inflammatory responses were reduced in TLR2 and TLR4 KO mice

**Fig 2:**
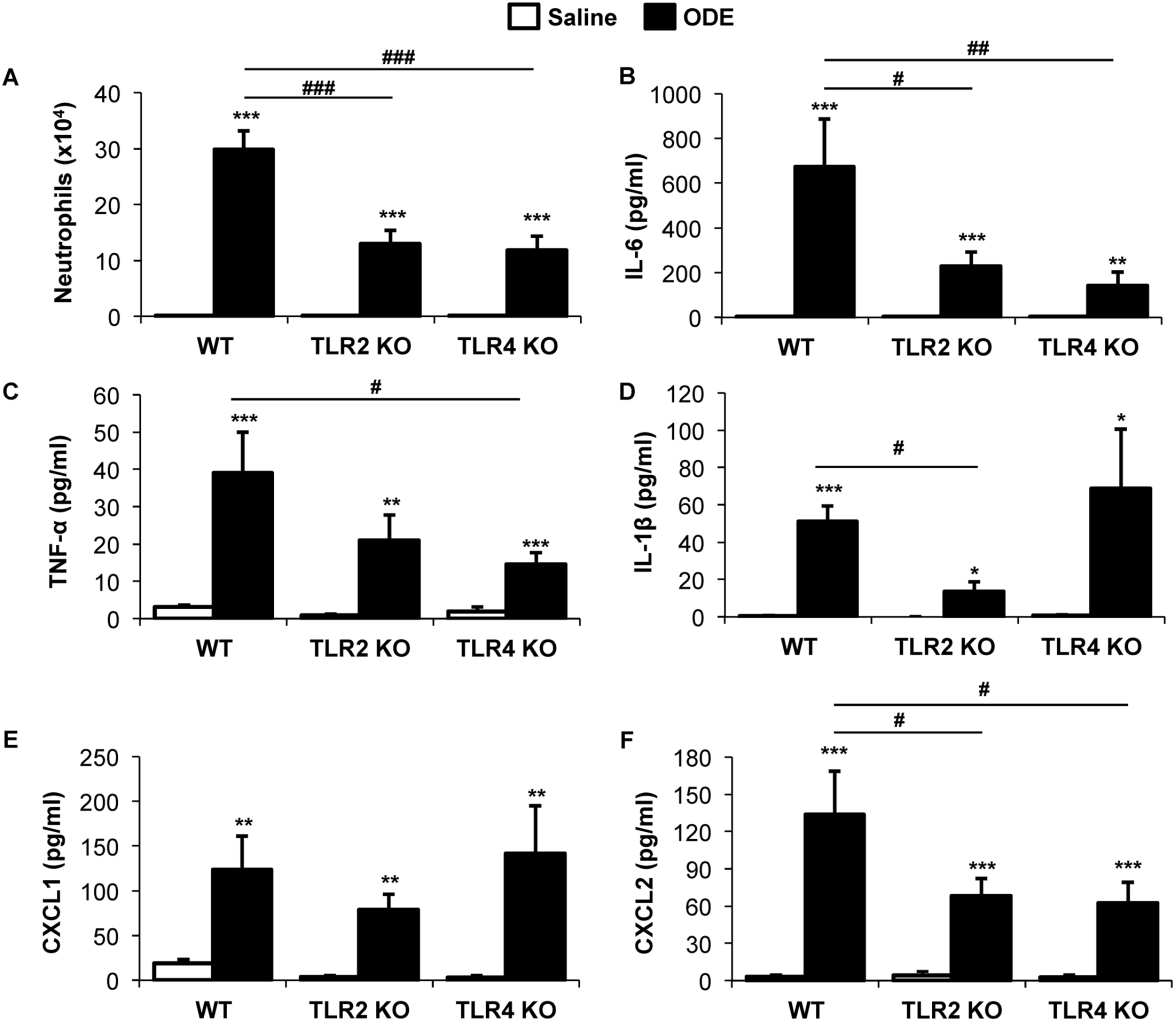
Repetitive inhalant organic dust extract (ODE)-induced airway inflammatory responses were reduced in TLR2 and TLR4 KO mice. WT, TLR2 KO, and TLR4 KO mice were *i*.*n*. treated with ODE daily or saline for 3 weeks whereupon animals were euthanized 5 hrs following final exposure. Neutrophil recruitment (**A**), IL-6 (**B**), TNF-α (**C**), IL-1β (**D**), CXCL1 (**E**), and CXCL2 (**F**) were determined in bronchoalveolar lavage fluid. Bar graphs represent the mean with standard error bars shown (N = minimum of 6 mice/group from 3 independent studies). Statistical significance denoted by asterisks (*p<0.01, **p<0.01, ***p<0.001) as compared to respective saline treatment group. Line denotes statistical significance (#p<0.05, ##p<0.01, ###p<0.001) of WT vs. TLR2 and TLR4 KO mice.

Repetitive, daily inhalant exposure to ODE for 3 weeks results in neutrophil recruitment and increased cytokine/chemokine release [30], and moreover, these airway inflammatory responses have been shown to be partially reduced in TLR2 and TLR4 KO mice [22,24,34]. In the current study, neutrophil influx induced by repetitive ODE treatments was significantly reduced (~50%) in TLR2 and TLR4 KO mice as compared to WT animals (Fig 2A). Moreover, there were reductions of ODE-induced cytokine and chemokine release to varying degrees in the TLR2 and TLR4 KO mice as compared to WT animals (Fig 2B–2F). Specifically, ODE-induced airway IL-6, IL-1β, and the murine neutrophil chemoattractant, CXCL2, levels were significantly reduced in TLR2 KO mice as compared WT animals. IL-6, TNF-α, and CXCL2 levels were significantly reduced in TLR4 KO mice as compared to WT animals. There was no difference in ODE-induced neutrophil influx and cytokine/chemokine release between TLR2 and TLR4 KO animals. These findings confirm earlier work by us and others demonstrating a role for TLR2 and TLR4 in mediating agriculture organic dust-induced airway inflammatory consequences.

### Repetitive inhalant ODE exposure-stimulated serum IL-6 levels are partially reduced in TLR2 and TLR4 KO mice

**Fig 3:**
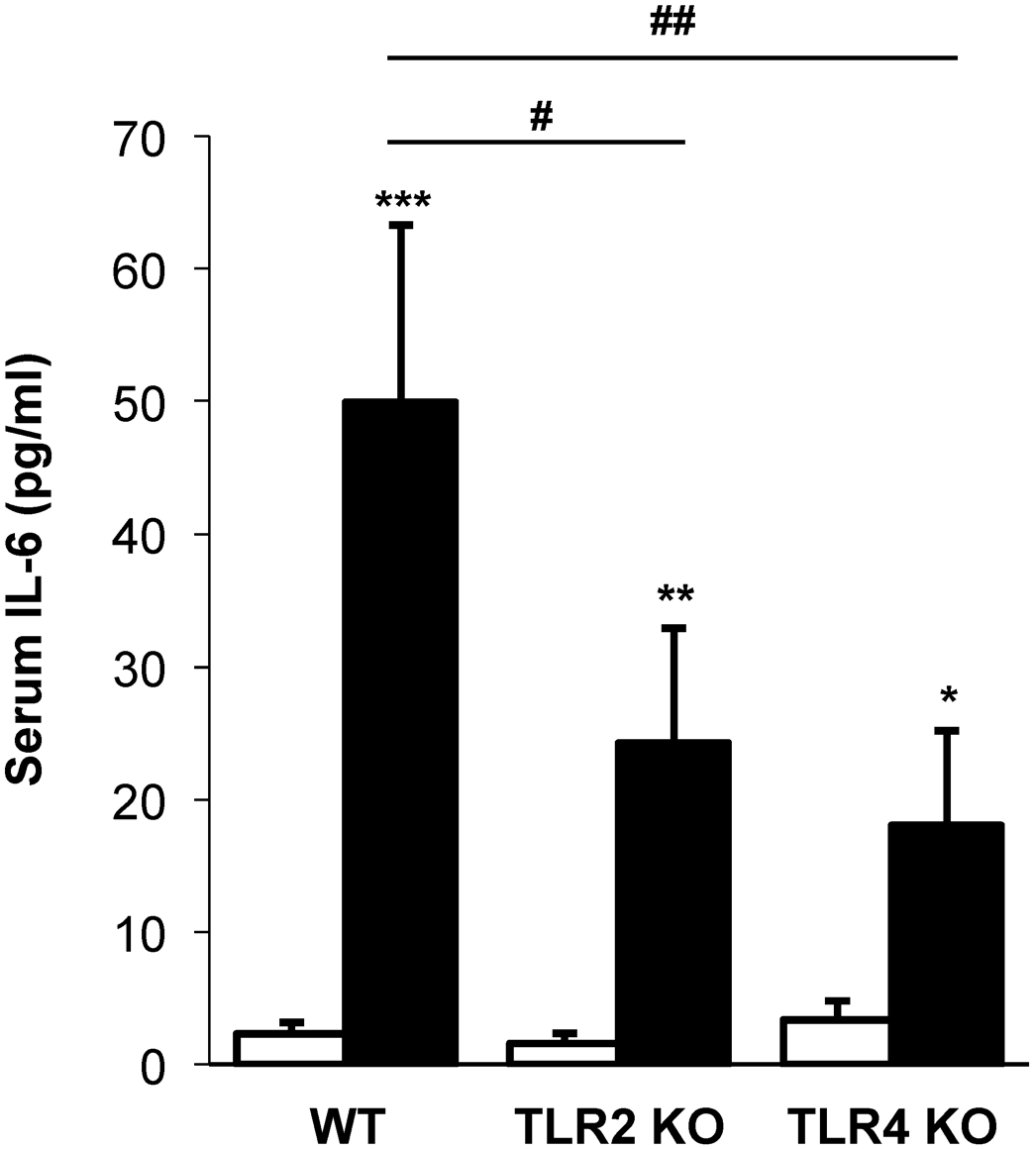
Repetitive inhalant ODE exposure-stimulated serum IL-6 levels are partially reduced in TLR2 and TLR4 KO mice. WT, TLR2 KO, and TLR4 KO mice were *i*.*n*. treated with ODE daily or saline for 3 weeks whereupon animals were euthanized 5 hrs following final exposure. Levels of IL-6 were determined in serum. Bar graphs represent the mean with standard error bars shown (N = minimum of 6 mice/group from 3 independent studies). Statistical significance denoted by asterisks (*p<0.05 **p<0.01, ***p<0.001) as compared to respective saline treatment group. Line denotes statistical significance (#p<0.05, ##p<0.01) of WT vs. TLR2 and TLR4 KO mice.

Daily, repetitive inhalant ODE exposure for 3 weeks resulted in increased serum IL-6 levels in WT, TLR2 KO and TLR4 KO mice; however, serum IL-6 levels were reduced in ODE-treated TLR2 KO and TLR4 KO mice as compared to ODE-treated WT animals (p<0.05; Fig 3). Serum levels of TNF-α, IL-1β, and IL-17 were below the lower limit of detection (data not shown).

### ODE-induced systemic bone loss is dependent upon TLR4, but not TLR2, signaling pathway

**Fig 4:**
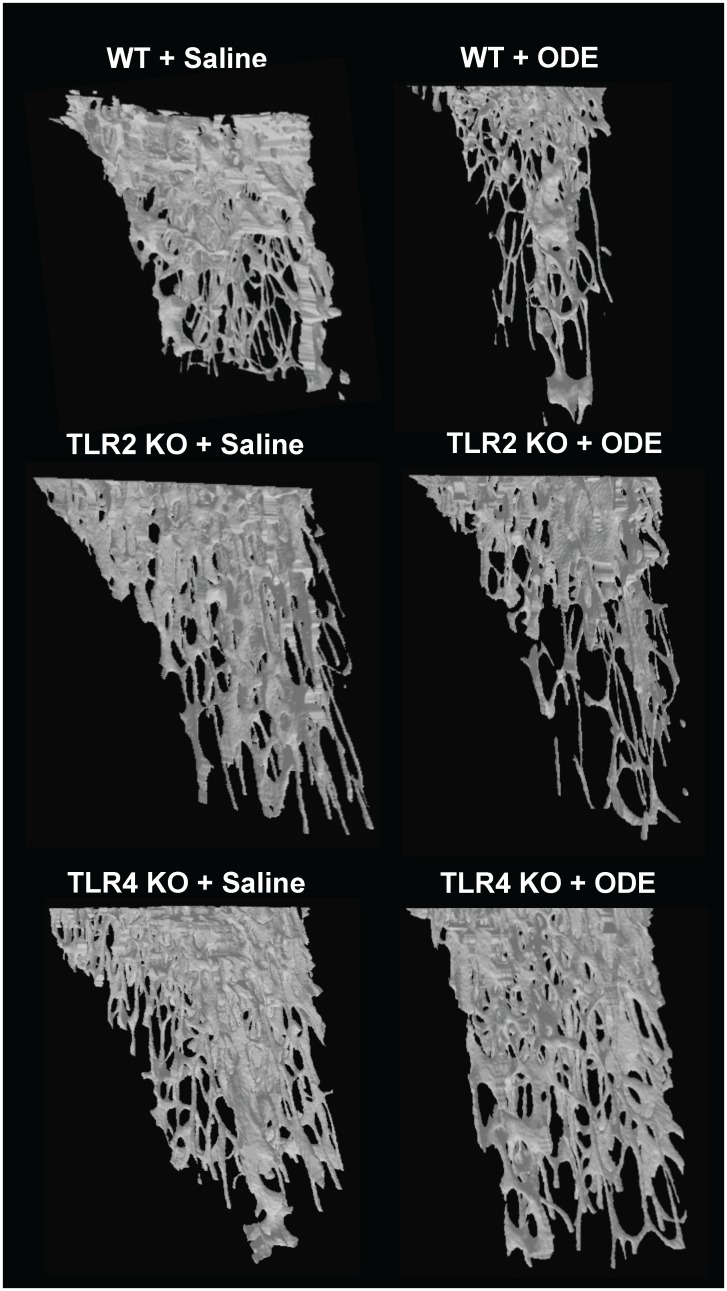
Loss of trabecular bone demonstrated in WT and TLR2 KO, but not TLR4 KO, mice treated repetitively with inhalant ODE. WT, TLR2 KO, and TLR4 KO animals were *i*.*n*. treated daily with saline or ODE for 3 weeks. A representative three-dimensional (3D) reconstructed image from region of interest of proximal tibia from one mouse per treatment group (minimum of 6 mice/group from 3 independent studies).

**Fig 5:**
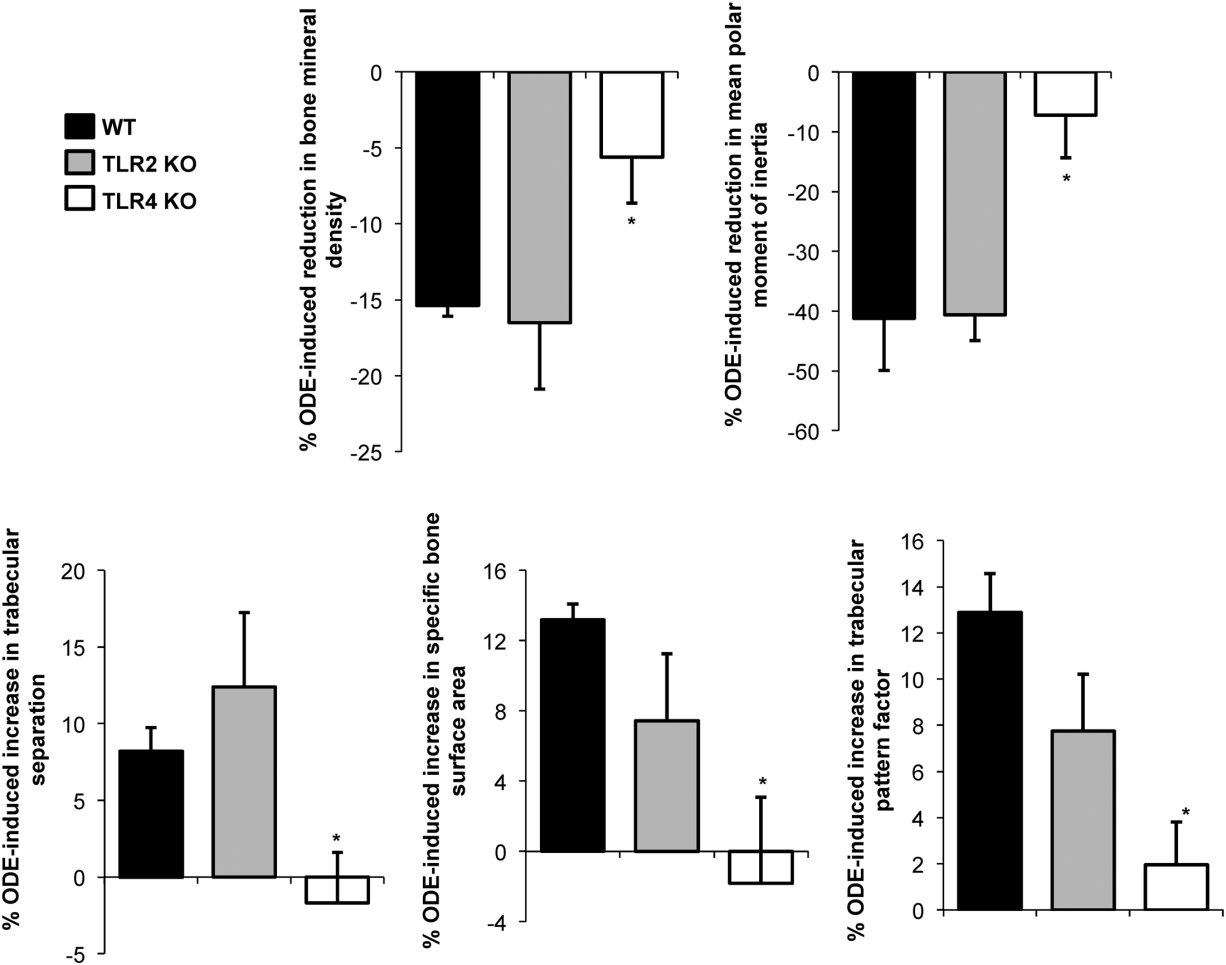
WT and TLR2 KO, but not TLR4 KO, mice were susceptible to the systemic bone deterioration response following repetitive ODE inhalation exposure by micro-CT analysis. WT, TLR4 KO, and TLR2 KO mice were *i*.*n*. treated daily with saline or ODE for 3 weeks whereupon trabecular bone of proximal tibia was analyzed micro-CT analysis. Changes in bone quality and bone quantity were observed in ODE-treated WT mice as compared to saline. TLR4 KO, but not TLR2 KO, animals were generally less responsiveness to bone changes induced by ODE. To compare findings across animal strains, bar graph depicts the mean with SEM bars of the percent change induced by ODE treatments (difference between ODE and saline treatment divided by saline groups multiplied by 100) in bone parameters compiled from three independent studies of 2–3 mice per study (N = 6–9 mice). Asterisks denote statistical significance (*p<0.05) vs. WT.

The tibias from the saline and ODE treated animals were investigated for bone quality and quantity by micro-CT imaging and analysis. Fig 4 depicts a representative 3D reconstructed image of the proximal tibia form one mouse per treatment group. Fig 5 demonstrates the quantitative changes in bone deterioration as a percentage of ODE-induced changes in bone parameters as compared to saline treatment for each animal strain (i.e. WT, TLR2 KO, TLR4 KO) with comparisons determined across animal strains. Consistent with previous studies [13], bone deterioration effects following repetitive inhalant ODE treatments as compared to saline treatments were demonstrated in this study in WT animals. TLR2 KO mice were also susceptible to ODE-induced bone deterioration; however, TLR4 KO animals demonstrated less ODE-induced bone deterioration. WT and TLR2 KO mice treated with inhalant ODE displayed an approximate 15% reduction in bone mineral density (Fig 5). Increases in trabecular separation, specific bone surface area, and trabecular pattern factor (all indicators of bone deterioration) were demonstrated in ODE treated WT and TLR2 KO mice (Fig 5). Inhalant ODE exposure also reduced the polar moment of inertia parameter by approximately 40% in both WT and TLR2 KO animals (Fig 5). There were no significant ODE-induced differences between WT and TLR2 KO mice for any of these measures. In contrast, as compared to WT animals, TLR4 KO mice were protected against ODE-induced bone deterioration parameters (p<0.05) including bone mineral density, trabecular separation, specific bone surface area, trabecular pattern factor, and mean polar moment of inertia (Fig 5).

### ODE exposure increased serum TRACP 5b levels in WT and TLR2 KO but not TLR4 KO animals

**Fig 6:**
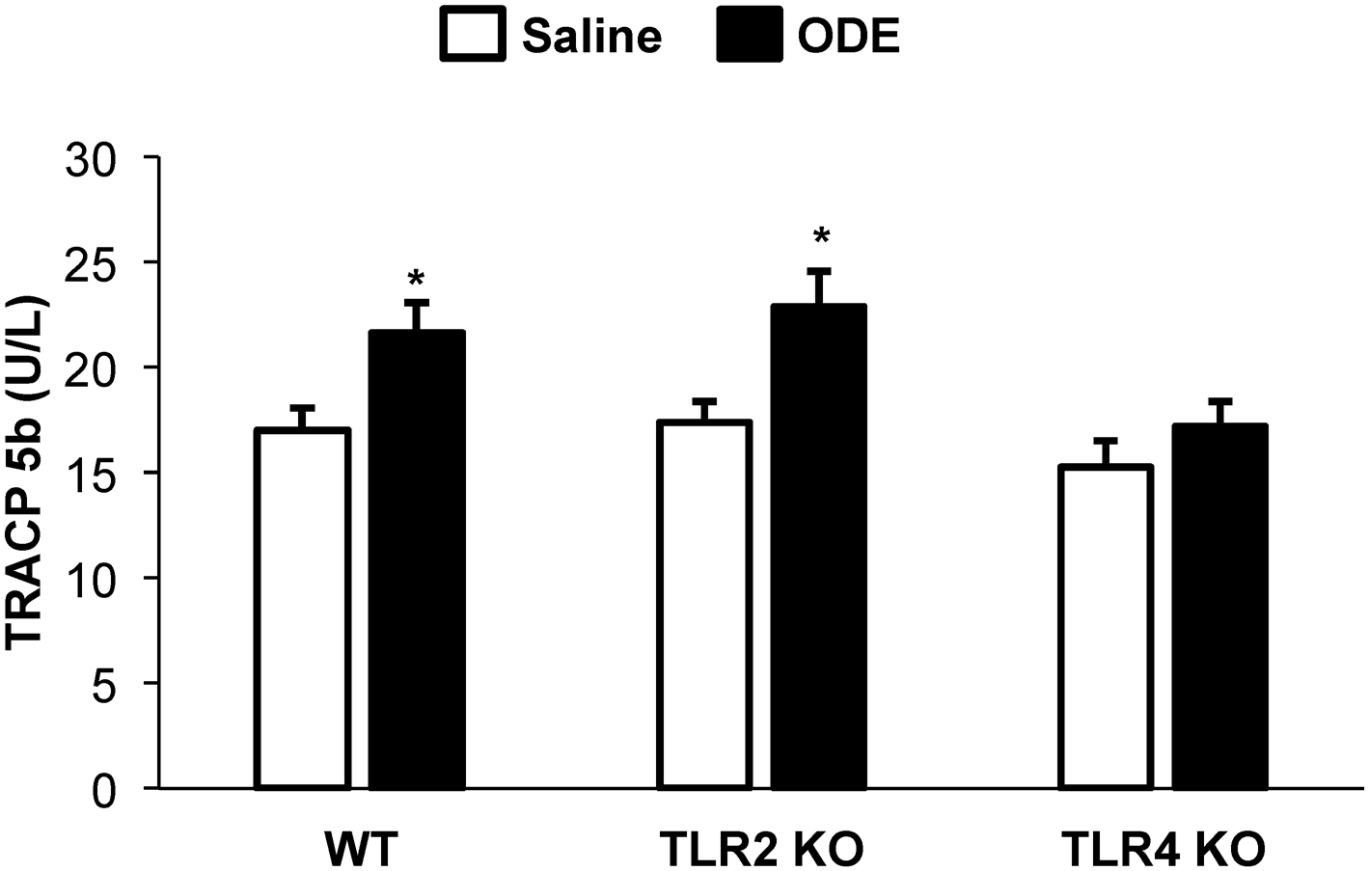
Repetitive inhalant ODE exposure increased serum TRACP 5b levels in WT and TLR2 KO but not TLR4 KO animals. WT, TLR2 KO, and TLR4 KO mice were *i*.*n*. treated with ODE daily or saline for 3 weeks whereupon animals were euthanized 5 hrs following final exposure. Levels of tartrate-resistant acid phosphatase 5b (TRACP 5b) were determined in serum. Bar graphs represent the mean with standard error bars shown (N = minimum of 6 mice/group from 3 independent studies). Statistical significance denoted by asterisks (*p<0.05) as compared to respective saline treatment group.

TRACP 5b, a serum marker of osteoclast number and bone resorption, was increased with repetitive ODE exposure in WT and TLR2 KO as compared to saline treated mice (p<0.05; Fig 6). However, there was no significant increase in serum TRACP 5b in TLR4 KO mice repetitively exposed to ODE as compared to saline treated mice (Fig 6).

### Repetitive inhalant ODE treatment increases osteoclast precursor populations in the murine bone marrow cells, which is dependent upon TLR4 signaling pathway

**Fig 7:**
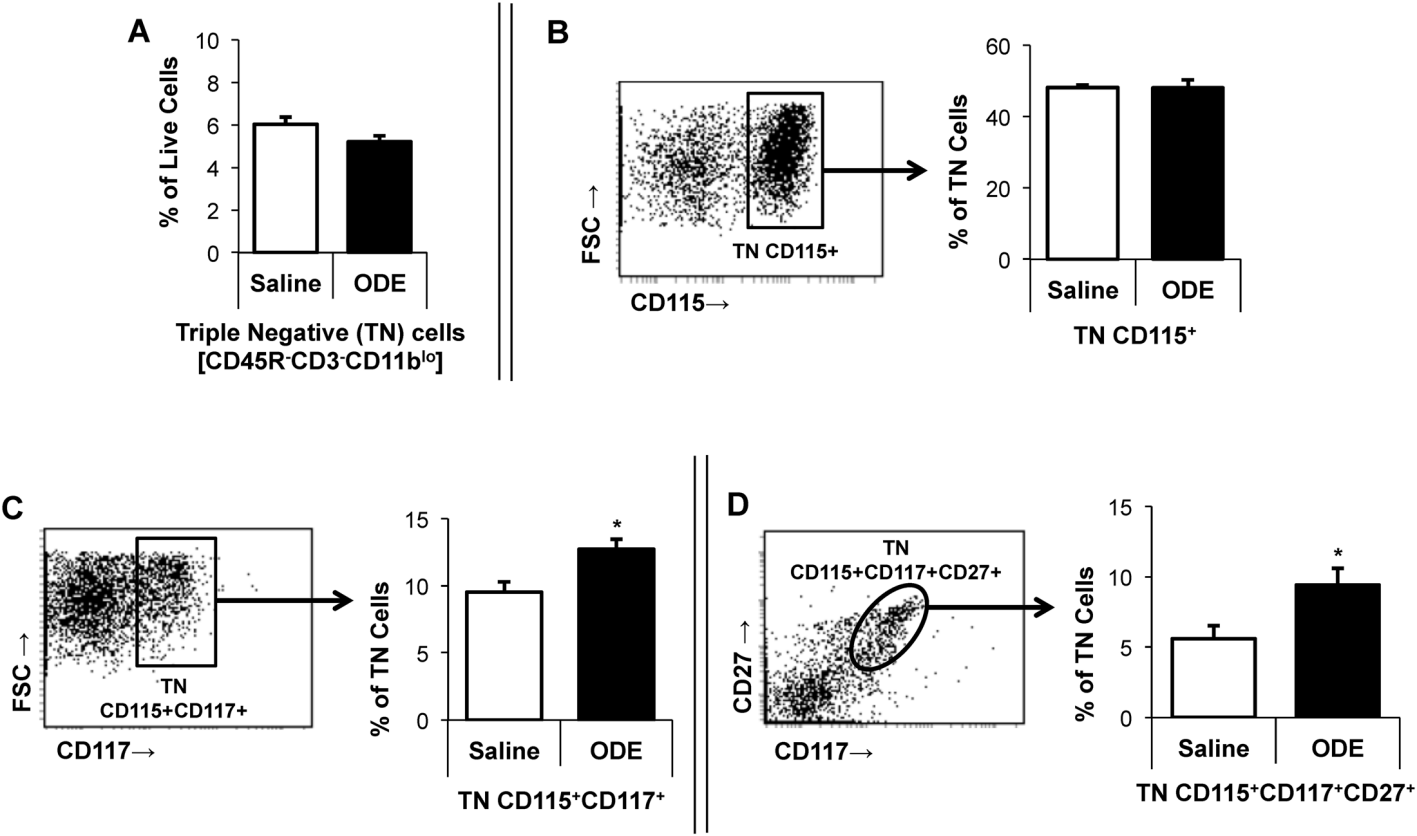
Inhalant exposure with organic dust extract (ODE) increases osteoclast precursor populations (OCP) in murine bone marrow cells. WT mice were *i*.*n*. treated with saline or ODE daily for three weeks whereupon mice were euthanized and bone marrow cells were collected and analyzed by flow cytometry. After exclusion of debris and dead cells, triple negative (TN) cells were gated based upon CD45R^-^CD3^-^CD11b^lo^ phenotype with **panel A** depicting the frequency of TN cells as a percentage of live cells in a bar graph. **Panel B** depicts a representative dot plot of TN cells expressing CD115 with associated frequency distribution shown in bar graph. Next, representative dot plots with frequency distribution of osteoclast precursor populations: TN CD115+CD117+ (**panel C**), and TN CD115+CD117+CD27+ (**panel D)** are shown. All bar graphs represent mean percentage with SEM bars. N = minimum of 6 mice/group from three independent experiments. Statistical significance denoted as asterisks (*p<0.05) vs. saline.

To determine whether repetitive inhalant ODE impacted osteoclast precursor (OCP) populations as a possible explanation for bone loss, OCP populations in murine marrow cells were identified by flow cytometry. In WT animals, there was no difference in the frequency of triple negative (TN; CD45R^-^CD3^-^CD11b^lo^) or TN/CD115^+^ early hematopoietic precursor cells between saline- and ODE-treatment (Fig 7).

**Fig 8:**
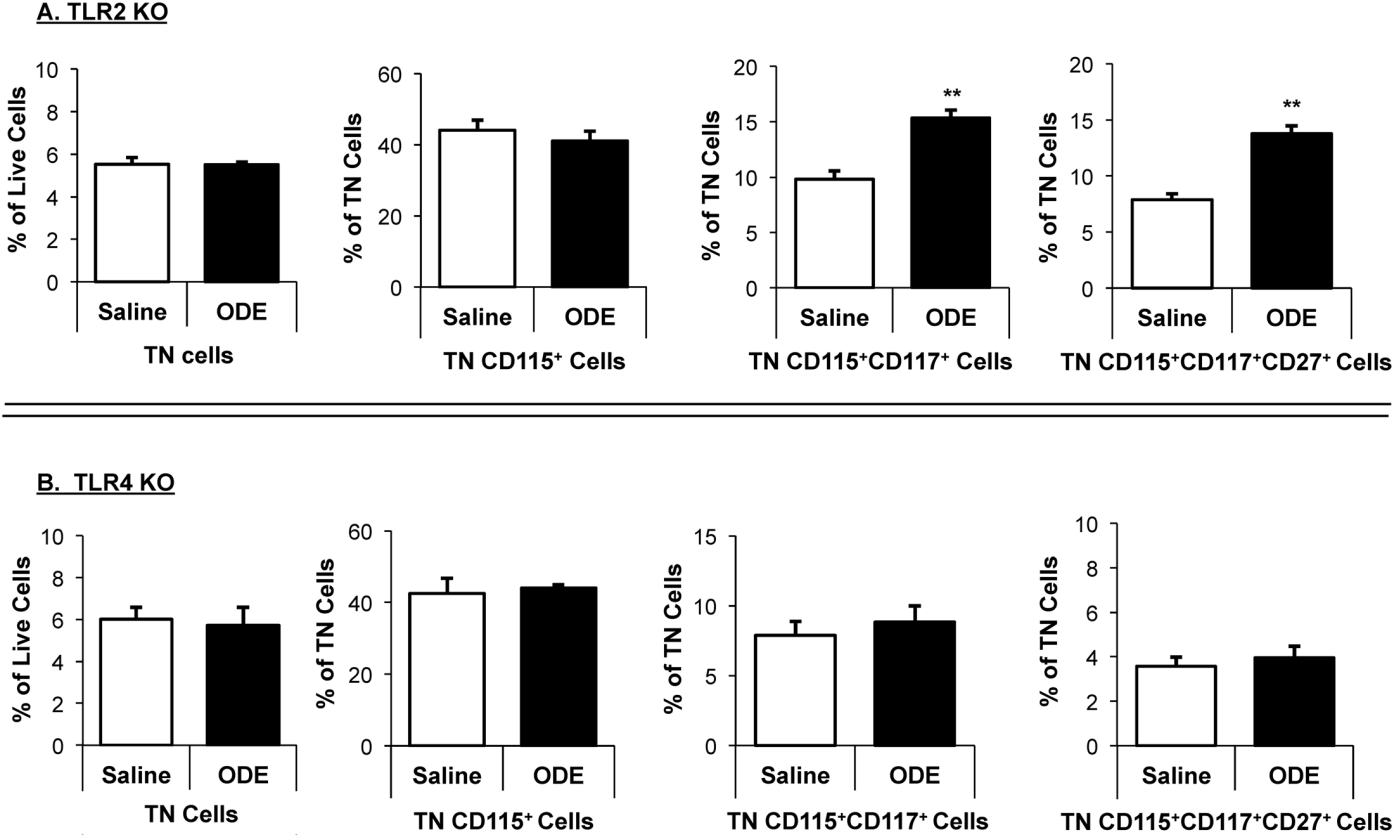
Increased osteoclast precursor populations (OCP) in murine bone marrow following inhalant ODE treatment is dependent upon TLR4, but not TLR2, signaling pathway. TLR2 KO (**A, top panel**) and TLR4 KO (**B, bottom panel**) mice were *i*.*n*. treated with saline or ODE daily for three weeks whereupon mice were euthanized and bone marrow cells were collected and analyzed by flow cytometry. After exclusion of debris and dead cells, triple negative (TN) cells were gated based upon CD45R^-^CD3^-^CD11b^lo^ phenotype. Distribution of TN cells are shown as mean percentage with SEM bars of live cells. Distribution of TN cells expressing CD115, CD115CD117, and CD115CD117CD27 are shown as mean percentage with SEM bars of TN cells. N = 4 mice/group. Statistical significance denoted by asterisks (**p<0.01) vs. saline.

However, compared to saline, ODE treatment significantly increased the frequency of TN/CD115^+^CD117^+^ as well as TN/CD115^+^CD117^+^CD27^+^ bone marrow OCP populations in both WT mice (p<0.05; Fig 7) and TLR2 KO mice (Fig 8). In contrast, there was no ODE-induced increase in bone marrow OCP populations in TLR4 KO mice (Fig 8).

## Discussion

In this study, inhalant ODE-induced bone deterioration was demonstrated to be dependent upon systemic TLR4, but not TLR2, signaling pathway despite similar contributions from both TLR2 and TLR4 pathways in mediating airway inflammatory consequences following ODE exposures. Specifically, TLR2 KO and TLR4 KO animals demonstrated an approximate 50% reduction in ODE-mediated airway neutrophil influx as well as various degrees of reduction in the magnitude of airway cytokine/chemokine release in response to ODE exposures. However, the TLR2 KO mice were as susceptible as the WT animals to the bone loss and disease manifestations induced by inhalant ODE exposures, which is in contrast to the TLR4 KO mice that were protected against ODE-mediated bone disease. Similarly, inhalant ODE exposures stimulated an increase in bone-resorbing osteoclasts and bone marrow osteoclast progenitor cells that was also TLR4 dependent as opposed to TLR2 dependent. Collectively, these findings suggest that the TLR4 recognition and signaling pathway is the dominant pathway responsible for mediating the systemic bone consequences following complex, microbial enriched organic dust inhalation exposures.

Microbial components are major drivers of agriculture-related organic dust-induced airway inflammation [9]. Endotoxin from cell walls of gram negative bacteria has been long-recognized as a potent inflammatory agent found within these organic dust environments [9]. However, there is not a universal association with levels of endotoxin within swine confinement facilities and respiratory health consequences because studies have reported high endotoxin exposure without airway symptoms [16]. Moreover, inhalation endotoxin challenges with 200-fold higher endotoxin concentrations than measured within swine barns did not reproduce similar human airway inflammatory responses as the actual swine barn exposure challenges [17]. Utilizing culture-dependent and culture-independent techniques, several studies have demonstrated a high burden of gram positive bacteria rather than gram negative bacteria, in organic dust samples from swine confinement buildings [18,20,41]. Previously, we demonstrated that exposure to gram positive peptidoglycan elicited airway inflammatory consequences in rodents similar to that observed with swine facility organic dust extracts, and that TLR2 signaling pathway was important in mediating airway inflammatory disease [22]. Importantly, TLR2 but not TLR4 gene polymorphisms were demonstrated by others to be associated with lung function among swine workers [21]. Consistent with these prior observations, our current study demonstrates that TLR2 signaling pathway remains important in explaining the airway inflammatory consequences to inhalant ODE exposures. However, a role for the TLR2 signaling pathway in mediating systemic bone disease was not found. In contrast, the inhalant ODE-induced bone consequences were mostly explained by the systemic TLR4 signaling pathway.

Both TLR2 and TLR4 ligands induce bone-resorbing osteoclast development (osteoclastogenesis) in a dose-dependent manner [25–28]. In proof-of-concept studies (Fig 1), we demonstrated that ODE, LPS (TLR4 ligand), and PGN (TLR2 ligand) enhanced osteoclastogenesis, but we found that i*n vitro* ODE-induced osteoclastogenesis was dependent upon TLR4 signaling pathway. Furthermore, it was demonstrated that osteoclastogenesis occurs in TLR2 KO and TLR4 KO cells with appropriate stimuli, indicating intact cellular machinery. However, it could be argued that the osteoclastogenesis assay does not reflect *in vivo* bioavailability. In our initial report describing bone loss and disease following repetitive inhalation exposure to complex ODE, comparison experiments were also conducted with inhalant LPS and PGN [13]. There was a divergence between lung parenchymal inflammation and degree of bone loss between LPS and PGN murine exposure groups. Namely, animals in the inhalant LPS treatment groups demonstrated only mild changes in lung histopathology, yet bone deterioration was greatest. In contrast, inhalant PGN exposures resulted in the greatest changes in lung histopathology, yet the least amount of comparable bone changes. Endotoxins are small, hydrophobic molecules that we suspect are likely to escape from the lung to potentially impact systemic manifestations of lung disease. In contrast, peptidoglycans are multilayered and cross-linked rigid structure that are not likely to be systemically disseminated from the lung [42]. Based upon this collective evidence, we suggest that endotoxin within ODE is released systemically and mediates bone loss consequences through engagement of the TLR4 signaling pathway.

Bone homeostasis is maintained as a result of the activities and cross-talk of bone-forming cells (osteoblasts) and bone-resorbing osteoclasts. TRACP 5b is an enzyme that serves as a marker of osteoclast number and bone resorption [43]. In this study we found that serum TRACP 5b levels were increased in ODE-treated WT animals, implicating osteoclasts as important in mediating ODE-induced bone loss. Moreover, this response was dependent on TLR4, not TLR2 signaling pathways. Osteoclasts are myeloid-derived cells, and using flow cytometry techniques, we further demonstrated that OCP cell population (TN CD115^+^CD117^+^CD27^+^) was increased in bone marrow cells collected from inhalant ODE-treated WT animals. Upregulation of OCPs represent a potential mechanism to explain the ODE-induced lung injury-bone loss connection. Moreover, ODE-induced OCPs were dependent upon TLR4, not TLR2 signaling pathway. This data correlates well with the micro-CT bone deterioration findings. It is also possible that osteoblasts are affected by inhalant ODE treatments, but studies to date have been unable to define this potential role (data not shown).

These studies emphasize the role for TLR4 ligand/receptor signaling pathway in mediating the lung-bone inflammatory axis to inhalant agriculture organic dust exposures. Potential applications include improving measures to reduce endotoxin exposure in these occupational settings and/or blocking TLR4 signaling. Another potential strategy might be to target downstream molecules induced by ODE-activated TLR4 signaling pathway. Downstream cytokine effectors such as IL-6, IL-17, and IL-1β have been implicated in promoting osteoclastogenesis [25,44]. Serum collected from exposed mice were screened for these specific cytokine responses, but only IL-6 was consistently detected. Because clinical approaches exist for blocking IL-6 [45,46], future studies could investigate the role of IL-6 in modulating bone deterioration induced by chronic airway inflammatory agents. Our studies are focused on the lung-bone axis because of the high prevalence of bone and airway disease in agricultural workers [9–11]. However, these data might have broader implications for understanding adverse systemic manifestations (e.g. cardiovascular disease [47]) associated with chronic inflammatory lung disease, for which additional studies would be necessary.

In summary, TLR2 and TLR4 pathways mediate complex ODE-induced airway inflammation, but bone deterioration following inhalant ODE exposures is strongly dependent upon TLR4. Inhalant ODE exposures significantly increased bone-resorbing osteoclast progenitors, which also directly depends on the presence of TLR4. Knowledge of how differing environmental inflammatory agents impact airway and subsequent bone consequences could be advantageous for development of preventative and/or therapeutic strategies to reduce disease burden in at-risk workers.

## Abbreviations

COPD: Chronic obstructive pulmonary disease
CT: Computed tomography
CXCL1: keratinocyte chemoattractant
CXCL2: macrophage inflammatory protein-2
IL: Interleukin
KO: Knockout
LPS: Lipopolysaccharide; endotoxin
M-CSF: Macrophage colony stimulating factor
mRANKL: membrane receptor activator of NF-κB ligand
OCP: Osteoclast precursors
ODE: Organic dust extract
PGN: Peptidoglycan
RANKL: Receptor activator of NF-κB ligand
TLR: Toll-like receptor
TNF-α: Tumor necrosis factor-alpha
WT: Wild type
